# Reinitiation and Subsequent Discontinuation of Angiotensin-Converting Enzyme Inhibitors and Angiotensin Receptor Blockers among New and Prevalent Users Aged 65 Years or More with Peripheral Arterial Disease

**DOI:** 10.3390/biomedicines11020368

**Published:** 2023-01-26

**Authors:** Martin Wawruch, Jan Murin, Tomas Tesar, Miriam Petrova, Martina Paduchova, Denisa Celovska, Beata Havelkova, Michal Trnka, Lucia Masarykova, Sofa D. Alfian, Emma Aarnio

**Affiliations:** 1 Institute of Pharmacology and Clinical Pharmacology, Faculty of Medicine, Comenius University, 811 08 Bratislava, Slovakia; 2 1st Department of Internal Medicine, Faculty of Medicine, Comenius University, 813 69 Bratislava, Slovakia; 3 Department of Organisation and Management of Pharmacy, Faculty of Pharmacy, Comenius University, 832 32 Bratislava, Slovakia; 4 Department of Angiology, Health Centre, 917 01 Trnava, Slovakia; 5 General Health Insurance Company, 851 04 Bratislava, Slovakia; 6 Institute of Medical Physics, Biophysics, Informatics and Telemedicine, Faculty of Medicine, Comenius University, 813 72 Bratislava, Slovakia; 7 Department of Pharmacology and Clinical Pharmacy, Faculty of Pharmacy, Universitas Padjadjaran, Jatinangor 45363, Indonesia; 8 Center of Excellence in Higher Education for Pharmaceutical Care Innovation, Universitas Padjadjaran, Jatinangor 45363, Indonesia; 9 School of Pharmacy, University of Eastern Finland, 70211 Kuopio, Finland

**Keywords:** peripheral arterial disease, non-persistence, reinitiation, ischemic stroke, myocardial infarction, new user, statins, antiplatelet agents

## Abstract

Angiotensin-converting enzyme inhibitors (ACEIs) or angiotensin-receptor blockers (ARBs) are recommended in the treatment of arterial hypertension in patients with peripheral arterial disease (PAD). The aims of our study were: (a) to analyse the extent of reinitiation and subsequent discontinuation in older hypertensive PAD patients non-persistent with ACEIs/ARBs; (b) to determine patient and medication factors associated with reinitiation and subsequent discontinuation; and (c) to compare these factors between prevalent and new users. The analysis of reinitiation was performed on a sample of 1642 non-persistent patients aged ≥65 years with PAD newly diagnosed in 2012. Patients reinitiating ACEIs/ARBs were used for the analysis of subsequent discontinuation identified according to the treatment gap period of at least 6 months without any prescription of ACEI/ARB. In the group of non-persistent patients, 875 (53.3%) patients reinitiated ACEIs/ARBs during a follow-up (24.8 months on average). Within this group, subsequent discontinuation was identified in 414 (47.3%) patients. Being a new user was associated with subsequent discontinuation, but not with reinitiation. Myocardial infarction during non-persistence and after reinitiation was associated with reinitiation and lower likelihood of subsequent discontinuation, respectively. Being a prevalent or a new user is associated with the use of medication also after initial discontinuation.

## 1. Introduction

According to the systematic review by Song et al. [1], 236.6 million people (≥25 years) were globally affected with peripheral arterial disease (PAD) in 2015, 72.9% of them living in low- and middle-income countries. The prevalence of PAD increases with age, and the prevalence in high-income countries has been reported to be around 5% at the age of 45–49 years, and 18–19% at the age of 85–89 years [2].

Arterial hypertension (along with smoking, diabetes, and hypercholesterolemia) represents one of the most important risk factors of PAD [1,2]. Management of PAD includes lipid-lowering and antiplatelet treatment, anticoagulation, peripheral vasodilators, antihypertensive therapy, control of diabetes mellitus, exercise therapy, and smoking cessation [3,4,5]. According to the European treatment guidelines, blood pressure is recommended to be controlled at <140/90 mmHg among patients with PAD and arterial hypertension, except for patients with diabetes mellitus, in whom a diastolic blood pressure of ≤85 mmHg is recommended, and old frail patients in whom recommended values of blood pressure should be achieved only if well tolerated without orthostatic hypotension. Angiotensin-converting enzyme inhibitors (ACEIs) or angiotensin-receptor blockers (ARBs) are recommended as the first-line treatment [6]. According to the ACC/AHA guideline, ACEIs and ARBs can be effective in reducing the risk of cardiovascular (CV) events in PAD patients [7].

Adequate patients’ adherence to medications recommended in the treatment of PAD is necessary for achieving benefits in these patients. Adherence has three components: initiation, implementation, and persistence. The process begins with initiation when the patient takes the first dose of the prescribed drug. Implementation reflects the extent to which a patient’s actual dosing regimen corresponds to the prescribed dosing regimen, from initiation until discontinuation, which represents the end of the process, when the patient stops taking the drug. Persistence refers to the time from treatment initiation to discontinuation [8,9,10].

In non-persistent patients, reinitiation and subsequent discontinuation of treatment represent a relatively common and important phenomenon, which has been identified, for example, in the case of statin treatment [11]. In the literature, there are no studies analysing the reinitiation and subsequent discontinuation of ACEI/ARB therapy in older patients with PAD. For this reason, the aims of our study presented in this manuscript were: (a) to analyse the extent of reinitiation and subsequent discontinuation in older hypertensive patients with PAD who discontinued ACEI/ARB therapy in our previous study [12]; (b) to determine associations between patient and medication factors and reinitiation and subsequent discontinuation of ACEIs/ARBs; and (c) to compare the extent of and factors associated with reinitiation and subsequent discontinuation between the groups of prevalent and new users of ACEIs/ARBs. The reason why we decided to compare the factors associated with reinitiation and subsequent discontinuation between prevalent and new users was the fact that new users of ACEIs/ARBs were at an increased probability of discontinuation in our previous study [12].

## 2. Materials and Methods

### 2.1. Database and Study Population

In our recently published retrospective cohort study, we analysed the discontinuation of ACEIs/ARBs in older PAD patients. The study cohort included 7080 older hypertensive patients aged ≥65 years (3075 men and 4005 women) diagnosed with PAD in 2012 and taking ACEIs/ARBs. The study cohort included both prevalent users (*n* = 6624) in whom ACEI/ARB treatment was initiated before PAD diagnosis and new users (*n* = 456) in whom ACEI/ARB therapy was started at the time of PAD diagnosis. In total, 1642 (23.2%) patients from the whole study cohort, 685 of them men (22.3%) and 957 women, (23.9%) discontinued therapy during the 5-year follow-up [12].

The analysis of reinitiation presented in this manuscript was performed on a sample of 1642 patients identified as non-persistent with ACEIs/ARBs in our previous study [12]. The data applied in our study were collected from the database of the largest health insurance company in Slovakia, the General Health Insurance Company. We did not have any direct access to the database of the General Health Insurance Company, which provided us with a database of PAD patients diagnosed in 2012. The derivation of the study cohort of older hypertensive PAD patients is described in detail in the previous manuscript [12].

### 2.2. Analysis of Reinitiation and Subsequent Discontinuation

Reinitiation was defined as the first use of ACEI/ARB observed after the period of non-persistence. After initial discontinuation determined in our previous study [12], patients were followed until reinitiation, the end of the 5-year follow-up period which started at the index date of our previous study (at the time of PAD diagnosis between 1 January and 31 December 2012 in patients treated with ACEIs/ARBs), or until patient´s death, whichever occurred first. The index date was the date of the initial discontinuation identified in our previous study [12].

Subsequent discontinuation was analysed in the group of patients who reinitiated ACEI/ARB treatment. Subsequent discontinuation was identified based on the presence of an at least 6-month treatment gap period without any prescription of ACEI/ARB, starting from the estimated date of the last day covered by the last package of the prescribed drug [12]. Patients with a 6-month treatment gap period were considered as non-persistent (i.e., subsequent discontinuation). Patients without such a gap period were classified as persistent. After reinitiation, patients were followed until subsequent discontinuation of ACEI/ARB treatment, until the end of the 5-year follow-up of our previous study [12], or until the date of their death, whichever occurred first. The index date of the study of the subsequent discontinuation was the date of reinitiation of ACEI/ARB treatment after the period of non-persistence.

Analyses of reinitiation/subsequent discontinuation were performed in the whole study cohort and separately in the groups of prevalent and new users. New users of ACEIs/ARBs were defined as patients in whom ACEI/ARB treatment was started at the time of PAD diagnosis. Patients in whom ACEI/ARB treatment was initiated before PAD diagnosis, i.e., those who were already treated with ACEIs/ARBs at the time of PAD diagnosis, were considered as prevalent users.

### 2.3. Factors Associated with Reinitiation/Subsequent Discontinuation of ACEI/ARB Treatment

Factors potentially associated with reinitiation/subsequent discontinuation of ACEI/ARB treatment included the same characteristics (socio-demographic characteristics, history of CV events, comorbid conditions, ACEI/ARB related characteristics, and CV co-medication) as those evaluated in the analysis regarding non-persistence in our previous study [12]. History of CV events (i.e., ischemic stroke, transient ischemic attack (TIA), and myocardial infarction (MI)) covered the 5-year period before the index date of the study of reinitiation/subsequent discontinuation. Additionally, CV events, which occurred during the period of non-persistence and the period of reinitiation, were evaluated as factors potentially associated with the likelihood of reinitiation and subsequent discontinuation, respectively. The association between the agent recorded as the last prescribed ACEI/ARB before initial discontinuation identified in our previous study [12] and reinitiation was also evaluated. The agent recorded as the first prescribed ACEI/ARB at the time of reinitiation was analysed as a factor potentially associated with the likelihood of subsequent discontinuation. Duration of the period of persistence before initial discontinuation analysed in our previous study [12] and the period of non-persistence after initial discontinuation were analysed as factors potentially associated with the likelihood of reinitiation and subsequent discontinuation, respectively.

### 2.4. Statistical Analysis

Continuous variables were expressed as means ± standard deviations and categorical variables were characterised as frequencies and percentages.

Categorical variables were compared between the two groups using the χ^2^-test. When the expected count was less than five in ≥20% of cells of the contingency table, the Fisher exact test was applied. To compare continuous variables between the two groups, the Mann-Whitney U test was used. The non-Gaussian distribution of evaluated variables was the reason why this non-parametric test was used. The normality of the distribution was analysed using the Kolmogorov-Smirnov test.

To compare the reinitiation and subsequent discontinuation between new and prevalent users of ACEIs/ARBs, the Kaplan-Meier model was used. Log-Rank, Breslow, and Tarone-Ware tests were used to identify any statistical significances in the difference in reinitiation and subsequent discontinuation between new and prevalent users.

The patient and medication characteristics potentially associated with the probability of reinitiation and subsequent discontinuation were identified using the Cox regression with time-dependent covariates. Ischemic stroke, TIA, and MI occurring during the period of non-persistence after initial discontinuation or after reinitiation represented time-dependent covariates. All other characteristics were time-independent covariates. Hazard ratios and corresponding 95% confidence intervals were determined for each evaluated characteristic [13]. 

All statistical tests were performed at the level of statistical significance of α = 0.05. Statistical software IBM SPSS for Windows, version 28, was used (IBM SPSS Inc., Armonk, NY, USA).

## 3. Results

The baseline characteristics of the whole cohort of non-persistent patients (*n* = 1642) are described in our previous manuscript [12]. The baseline characteristics of reinitiating patients and those who did not reinitiate, as well as characteristics of reinitiators who were persistent or became non-persistent after reinitiation are provided in Table 1.

Among non-persistent patients (*n* = 1642), 875 (53.3%) patients reinitiated ACEIs/ARBs during a follow-up (24.8 months on average). Of them, 754 (86.2%) were prevalent users and 121 (13.8%) were new users. The group of 767 non-reinitiating patients consisted of 697 (90.9%) prevalent users and 70 (9.1%) new users of ACEIs/ARBs. Baseline characteristics of reinitiators/non-reinitiators in the groups of prevalent and new users are shown in Supplementary Table S1.

Within the group of 875 reinitiating patients, non-persistence (subsequent discontinuation) was identified in 414 (47.3%) patients. This group included 350 (84.5%) prevalent users and 64 (15.5%) new users. The group of 461 persistent patients included 404 (87.6%) prevalent users and 57 (12.4%) new users. Baseline characteristics of patients who discontinued and those who did not discontinue ACEI/ARB treatment after reinitiation in the groups of prevalent and new users are shown in Supplementary Table S2.

**Figure 1:**
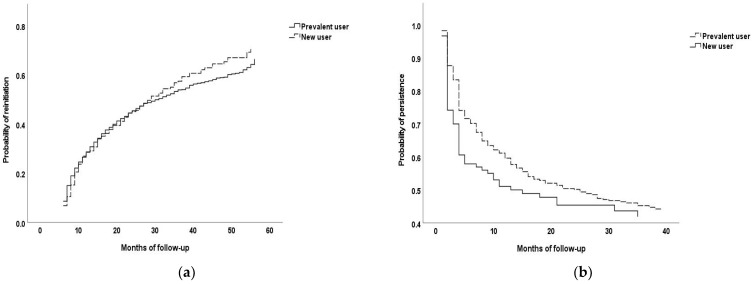
Kaplan–Meier curves of (**a**) reinitiation of ACEI/ARB treatment, and (**b**) subsequent discontinuation among reinitiating patients.

Reinitiation was compared between prevalent and new users in the Kaplan–Meier model (Figure 1a). We did not find any significant difference in the probability of reinitiation between these two groups (*p* = 0.428 according to the Log-Rank test; *p* = 0.988 according to the Breslow test; and *p* = 0.707 according to the Tarone–Ware test). On the other hand, in the comparison of the probability of non-persistence between prevalent and new users (Figure 1b), the curve of new users declined more steeply than that of prevalent users. According to the Breslow test (*p* = 0.009) and Tarone–Ware test (*p* = 0.029), there was a significant difference. However, there was no significant difference according to the Log-Rank test (*p* = 0.110).

Based on our Cox regressions in the whole cohort and the two subgroups, factors increasing the probability of reinitiation included MI during non-persistence (whole cohort and prevalent users), history of MI (new users), and administration of irbesartan (new users) (Table 2). On the other hand, in the whole study cohort and in the subgroup of prevalent users, a longer duration of persistence before initial discontinuation was associated with a decreased likelihood of reinitiation.

Ischemic stroke after reinitiation (whole cohort and prevalent users), administration of ramipril, losartan, and antiplatelet agents (new users), and being a new user of ACEI/ARB therapy (whole cohort), were associated with an increased probability of subsequent discontinuation after reinitiation. On the other hand, history of ischemic stroke (whole cohort and prevalent users), MI after reinitiation (whole cohort), and administration of statins (whole cohort) represented factors associated with a decreased likelihood of subsequent discontinuation in reinitiating patients.

## 4. Discussion

In the study presented in this manuscript, the reinitiation of ACEI/ARB treatment was identified in more than half of 1642 older hypertensive PAD patients who discontinued this treatment during the 5-year follow-up in our previous study [12]. However, almost one half of the 875 reinitiating patients discontinued the ACEI/ARB treatment again. These results indicate a relatively common stop-starting behaviour in older hypertensive PAD patients taking ACEIs/ARBs. A large proportion of patients who discontinued ACEI/ARB treatment again after reinitiation may suggest an insufficient awareness of the significance of this therapy in hypertensive PAD patients. This behaviour was also described by Vinogradova et al. [11] in their cohort study, which analysed the discontinuation and restarting of statin treatment. Si et al. [14] reported the reinitiation of ACEIs in 33% and ARBs in 43% of patients among older Australians. In the study by Alfian et al. [15] of 1201 patients who discontinued antihypertensive drugs in the first year, 22% reinitiated therapy within one year. Their cohort study evaluated the predictors of non-adherence, non-persistence and reinitiation of blood pressure-lowering medication among patients taking oral antidiabetic medications in the Netherlands. According to the retrospective cohort study involving new users of antihypertensive drugs by van Wijk et al. [16], 19% of 18,357 patients who discontinued treatment restarted it within one year, and 61% restarted it within six years. In the retrospective population-based study by Mahmoudpour et al. [17], the prescription patterns of ACEIs for various indications (arterial hypertension, heart failure, MI, and renal disease) were evaluated. Non-persistent patients were identified according to a 6-month treatment gap period, and a restart of ACEIs was reported in 18% of non-persistent hypertensive patients.

**Table 2:** Multivariate analysis of the association between patient- and medication-related characteristics and the likelihood of reinitiation/subsequent discontinuation after reinitiation among prevalent and new users.

	Analysis of Reinitiation			Analysis of Subsequent Discontinuation		
	The Whole Study Cohort (*n* = 1642)	Prevalent Users (*n* = 1451)	New Users (*n* = 191)	The Whole Study Cohort (*n* = 875)	Prevalent Users (*n* = 754)	New Users (*n* = 121)
*Socio-demographic characteristics*						
Age	1.00 (0.99–1.01)	1.00 (0.98–1.01)	0.98 (0.92–1.03)	0.99 (0.97–1.01)	1.00 (0.98–1.02)	0.94 (0.85–1.03)
Female sex	0.87 (0.75–1.01)	0.90 (0.76–1.05)	0.79 (0.43–1.43)	0.97 (0.77–1.21)	0.98 (0.77–1.25)	0.66 (0.24–1.80)
University education	1.03 (0.79–1.35)	0.98 (0.73–1.31)	1.82 (0.71–4.65)	0.72 (0.47–1.11)	0.76 (0.47–1.21)	0.44 (0.10–2.08)
Employed patients	0.99 (0.71–1.37)	0.95 (0.67–1.35)	1.03 (0.34–3.13)	1.16 (0.74–1.81)	1.33 (0.82–2.15)	0.48 (0.08–3.07)
*History of CV events* ^a^						
History of ischemic stroke	0.98 (0.82–1.17)	0.96 (0.80–1.16)	1.12 (0.49–2.55)	**0.77 (0.60–0.98)**	**0.73 (0.56–0.95)**	1.01 (0.37–2.75)
History of TIA	0.94 (0.74–1.19)	0.95 (0.74–1.22)	0.70 (0.28–1.77)	0.82 (0.59–1.14)	0.73 (0.50–1.06)	1.34 (0.43–4.23)
History of MI	0.97 (0.76–1.22)	0.88 (0.68–1.14)	**3.10 (1.30–7.35)**	1.11 (0.80–1.53)	1.09 (0.77–1.54)	1.04 (0.32–3.40)
*CV events during non-persistence/the period of reinitiation*						
Ischemic stroke during non-persistence/the period of reinitiation	0.95 (0.69–1.30)	0.94 (0.67–1.33)	1.16 (0.43–3.13)	**1.55 (1.01–2.38)**	**1.70 (1.09–2.66)**	0.17 (0.01–2.56)
TIA during non-persistence/the period of reinitiation	1.14 (0.76–1.73)	1.07 (0.67–1.69)	0.97 (0.30–3.19)	1.41 (0.51–3.94)	1.31 (0.47–3.69)	
MI during non-persistence/the period of reinitiation	**1.64 (1.12–2.39)**	**1.65 (1.11–2.46)**	1.10 (0.21–5.79)	**0.38 (0.15–0.95)**	0.46 (0.19–1.15)	0.72 (0.30–3.12)
*Comorbid conditions*						
Number of comorbid conditions	0.96 (0.84–1.10)	0.96 (0.83–1.11)	0.82 (0.46–1.46)	1.09 (0.90–1.32)	1.10 (0.89–1.35)	0.94 (0.41–2.16)
Chronic heart failure	1.11 (0.80–1.53)	1.10 (0.78–1.55)	1.76 (0.51–6.00)	0.78 (0.48–1.28)	0.75 (0.44–1.29)	0.33 (0.03–3.80)
Atrial fibrillation	1.14 (0.86–1.52)	1.18 (0.87–1.60)	0.33 (0.08–1.42)	1.10 (0.72–1.67)	1.20 (0.77–1.87)	1.03 (0.11–9.45)
Diabetes mellitus	0.96 (0.78–1.19)	0.92 (0.73–1.15)	1.36 (0.61–3.02)	0.90 (0.67–1.21)	0.83 (0.60–1.15)	1.82 (0.56–5.89)
Hypercholesterolemia	1.07 (0.87–1.32)	1.07 (0.86–1.33)	1.04 (0.43–2.51)	0.78 (0.58–1.06)	0.76 (0.55–1.05)	0.85 (0.23–3.07)
Dementia	1.19 (0.87–1.64)	1.17 (0.84–1.63)	1.68 (0.36–7.79)	0.95 (0.60–1.50)	0.91 (0.55–1.49)	0.96 (0.16–5.93)
Depression	1.02 (0.78–1.32)	0.97 (0.74–1.29)	0.93 (0.30–2.90)	0.96 (0.65–1.42)	1.09 (0.73–1.64)	0.19 (0.03–1.18)
Anxiety disorders	1.05 (0.85–1.30)	1.07 (0.86–1.34)	0.97 (0.33–2.83)	0.96 (0.70–1.32)	0.97 (0.69–1.35)	1.15 (0.25–5.32)
Parkinson’s disease	1.37 (0.95–1.99)	1.23 (0.82–1.85)	3.46 (0.91–13.12)	1.05 (0.64–1.72)	1.01 (0.58–1.74)	2.92 (0.41–20.65)
Epilepsy	1.28 (0.84–1.94)	1.35 (0.88–2.06)	0.82 (0.28–3.32)	0.92 (0.50–1.68)	0.97 (0.53–1.77)	
Bronchial asthma/COPD	0.94 (0.75–1.18)	0.93 (0.73–1.19)	2.03 (0.75–5.50)	1.16 (0.84–1.61)	1.16 (0.81–1.64)	3.71 (0.81–17.12)
*ACEI/ARB related characteristics*						
ACEI/ARB agent ^b^						
Perindopril	1.00	1.00	1.00	1.00	1.00	1.00
Lisinopril	0.87 (0.62–1.22)	0.86 (0.60–1.25)	1.26 (0.37–4.28)	1.28 (0.70–2.33)	1.02 (0.54–1.91)	7.93 (0.27–17.72)
Ramipril	1.07 (0.86–1.32)	1.12 (0.89–1.41)	0.55 (0.26–1.18)	0.89 (0.64–1.24)	0.73 (0.51–1.06)	**4.35 (1.23–15.35)**
Enalapril	0.52 (0.19–1.41)	0.53 (0.20–1.44)		3.40 (0.77–15.01)	3.41 (0.77–15.17)	
Spirapril	0.88 (0.12–6.44)	0.90 (0.12–6.62)		0.95 (0.13–6.95)	0.95 (0.13–6.99)	
Trandolapril	0.95 (0.77–1.17)	0.98 (0.78–1.23)	0.81 (0.39–1.69)	0.99 (0.74–1.32)	1.02 (0.74–1.40)	0.86 (0.30–2.44)
Quinapril	1.21 (0.92–1.60)	1.29 (0.97–1.73)	0.60 (0.15–2.35)	0.69 (0.41–1.16)	0.62 (0.37–1.05)	
Imidapril	0.75 (0.42–1.34)	0.75 (0.41–1.39)	0.78 (0.05–11.40)	1.27 (0.39–4.16)	1.22 (0.37–4.01)	
Fosinopril	0.49 (0.20–1.19)	0.50 (0.20–1.21)		1.55 (0.37–6.59)	1.61 (0.38–6.88)	
Valsartan	1.07 (0.75–1.55)	1.11 (0.77–1.60)	0.71 (0.22–3.02)	1.21 (0.64–2.26)	1.20 (0.64–2.26)	
Losartan	1.33 (0.83–2.13)	1.27 (0.78–2.06)	8.03 (0.43–15.55)	1.60 (0.68–3.76)	1.23 (0.49–3.11)	**8.85 (2.22–18.34)**
Telmisartan	1.58 (0.97–2.56)	1.56 (0.95–2.57)	3.91 (0.84–9.82)	1.32 (0.60–2.90)	0.96 (0.41–2.26)	0.54 (0.01–4.80)
Candesartan	0.84 (0.44–1.58)	0.86 (0.45–1.63)		0.62 (0.20–2.98)	0.67 (0.23–3.04)	
Irbesartan	1.42 (0.58–3.50)	0.84 (0.21–3.46)	**6.41 (1.09–14.71)**	1.08 (0.26–4.50)	1.12 (0.27–4.67)	
Patient´s co-payment (EUR) ^c^	1.01 (0.99–1.03)	1.01 (0.99–1.03)	0.96 (0.88–1.04)	1.00 (0.97–1.04)	0.99 (0.96–1.03)	1.14 (0.99–1.30)
New ACEI/ARB agent user ^d^	1.04 (0.82–1.31)			**1.41 (1.02–1.95)**		
General practitioner as index prescriber	1.05 (0.88–1.24)	1.00 (0.84–1.20)	1.68 (0.92–3.05)	0.88 (0.69–1.12)	0.90 (0.69–1.18)	1.29 (0.55–2.98)
*CV co-medication*						
Number of medications	1.01 (0.98–1.05)	1.02 (0.99–1.06)	0.96 (0.83–1.12)	0.98 (0.94–1.03)	0.97 (0.92–1.03)	1.09 (0.88–1.34)
Number of CV medications	0.95 (0.88–1.02)	0.94 (0.87–1.01)	1.14 (0.83–1.55)	1.06 (0.95–1.19)	1.11 (0.99–1.26)	0.72 (0.43–1.20)
Antiplatelet agents	0.95 (0.80–1.13)	0.97 (0.81–1.16)	0.81 (0.39–1.69)	1.02 (0.81–1.30)	0.97 (0.75–1.25)	**3.42 (1.18–9.94)**
Anticoagulants	0.91 (0.74–1.11)	0.97 (0.78–1.20)	0.57 (0.25–1.28)	0.84 (0.62–1.12)	0.83 (0.61–1.14)	0.75 (0.21–2.67)
Cardiac glycosides	1.03 (0.74–1.42)	1.01 (0.71–1.42)	3.25 (0.76–13.93)	1.13 (0.70–1.82)	1.11 (0.66–1.87)	1.53 (0.12–19.65)
Antiarrhythmic agents	1.03 (0.75–1.42)	1.04 (0.74–1.45)	3.22 (0.67–15.36)	1.18 (0.73–1.89)	0.94 (0.57–1.57)	2.90 (0.28–21.93)
Beta-blockers	1.22 (0.99–1.49)	1.20 (0.97–1.48)	1.06 (0.46–2.45)	0.85 (0.63–1.15)	0.90 (0.65–1.25)	1.16 (0.35–3.88)
Thiazide diuretics	1.07 (0.88–1.31)	1.08 (0.87–1.33)	1.01 (0.44–2.31)	0.97 (0.72–1.30)	0.96 (0.70–1.31)	1.35 (0.40–4.52)
Loop diuretics	0.98 (0.78–1.24)	0.98 (0.77–1.24)	1.04 (0.40–2.71)	0.82 (0.58–1.15)	0.70 (0.48–1.01)	3.89 (0.75–20.25)
Mineralocorticoid receptor antagonists	0.98 (0.71–1.37)	1.02 (0.72–1.43)	0.35 (0.06–1.99)	0.83 (0.48–1.42)	0.77 (0.44–1.36)	3.71 (0.12–14.88)
Calcium channel blockers	1.02 (0.85–1.23)	1.04 (0.86–1.26)	0.41 (0.16–1.06)	0.89 (0.67–1.19)	0.76 (0.56–1.04)	2.25 (0.41–12.33)
Statins	1.18 (0.98–1.41)	1.17 (0.97–1.42)	1.01 (0.47–2.17)	**0.77 (0.60–0.99)**	0.78 (0.59–1.02)	0.45 (0.14–1.42)
Lipid-lowering agents other than statins ^e^	0.99 (0.76–1.28)	0.99 (0.75–1.30)	1.58 (0.56–4.45)	0.88 (0.60–1.30)	0.91 (0.61–1.36)	0.45 (0.10–2.09)
Duration of persistence/non-persistence (months) ^f^	**0.98 (0.97–0.99)**	**0.98 (0.97–0.99)**	1.02 (0.99–1.04)	1.00 (0.99–1.01)	1.00 (0.99–1.01)	1.00 (0.97–1.03)

Values represent hazard ratios (95% confidence intervals). In the case of statistically significant results (*p* < 0.05), the values are expressed in bold. TIA—transient ischemic attack; MI—myocardial infarction; COPD—chronic obstructive pulmonary disease; CV—cardiovascular. ^a^ The time period covered by “history”—5 years before the index date of the analysis of reinitiation/analysis of the subsequent discontinuation after reinitiation. ^b^ ACEI/ARB agent—in the analysis of reinitiation: the last ACEI/ARB agent before initial discontinuation/in the analysis of subsequent discontinuation after reinitiation: the ACEI/ARB agent administered initially at the time of reinitiation. ^c^ Patient´s co-payment—calculated as the cost of ACEI/ARB treatment paid by the patient per month; in the analysis of reinitiation: co-payment for the last ACEI/ARB agent before initial discontinuation/in the analysis of subsequent discontinuation after reinitiation: co-payment for the ACEI/ARB agent administered initially at the time of reinitiation. ^d^ New ACEI/ARB agent user—patient in whom ACEI/ARB treatment was initiated in association with the diagnosis of peripheral arterial disease. ^e^ Lipid-lowering agents other than statins—ezetimibe and fibrates. ^f^ In the analysis of reinitiation: duration of persistence before initial discontinuation/in the analysis of subsequent discontinuation after reinitiation: duration of the period of non-persistence (before reinitiation).

Being a new user of the ACEI/ARB treatment was associated with an increased probability of subsequent discontinuation after reinitiating treatment. Being a new user was also associated with an increased probability of initial discontinuation after the index date of our previous study [12]. In the case of initial discontinuation, this finding may be explained by the potentially increased risk of adverse drug reactions, which may occur at the beginning of the treatment. However, it is not possible to use this explanation in the case of subsequent discontinuation in reinitiating patients past the vulnerable period at the beginning of the treatment. This result may indicate a generally increased tendency to discontinue ACEI/ARB treatment in patients in whom this treatment was started at the time of PAD diagnosis (new users). This result suggests an insufficient awareness of the importance of ACEIs/ARBs when treating arterial hypertension in older PAD patients who are new users of these medications. According to the study by Si et al. [14], the risk of discontinuation is increased in the first 6 to 12 months of treatment and, similarly, Alfian et al. [15] concluded that the first year after starting treatment is the most critical in terms of discontinuation.

The history of ischemic stroke was associated with a decreased probability of subsequent discontinuation of ACEIs/ARBs in the whole cohort of reinitiating patients and in the subgroup of prevalent users. ACEIs/ARBs are recommended in the treatment of arterial hypertension in patients after stroke/TIA to prevent recurrent stroke [18]. This result may indicate that physicians correctly use ACEIs/ARBs in the treatment of hypertension in stroke patients and that these patients understand the importance of ACEIs/ARBs in the treatment of their hypertension after reinitiation. On the other hand, in our study, acute ischemic stroke after reinitiation was associated with an increased likelihood of subsequent discontinuation in the whole cohort of reinitiating patients and in the subgroup of prevalent users. A possible explanation of this result may be the discouraging effect of acute ischemic stroke on patients reinitiating ACEI/ARB treatment, who may consider this treatment as ineffective in preventing acute CV events. However, the design of our study does not make it possible to explain the divergent effects of a history of ischemic stroke vs. acute ischemic stroke during the period of reinitiation on the probability of subsequent discontinuation.

Acute MI during the period of non-persistence was associated with an increased likelihood of reinitiation in the whole study cohort and in the subgroup of prevalent users. MI after reinitiation decreased the probability of subsequent discontinuation in the whole cohort. MI represents a condition whose secondary prevention also requires administration of ACEIs/ARBs [19]. For this reason, this acute CV event may have stimulated reinitiation of ACEI/ARB treatment and prevented discontinuation of ACEIs/ARBs in reinitiating patients. History of MI was associated with an increased likelihood of reinitiation, but only in the subgroup of new users. This result may suggest that physicians use the history of this CV event as a supportive argument for encouraging non-persistent patients to reinitiate the ACEI/ARB treatment.

A longer period of persistence before the initial discontinuation was associated with a decreased probability of reinitiation in the whole cohort and also in the subgroup of prevalent users. It is possible that older hypertensive patients with PAD consider ACEI/ARB therapy to be useless after a certain period of treatment and do not reinitiate it. This may be caused, for example, by a deficient awareness of the beneficial effects of treatment. On the other hand, in the study by Alfian et al. [15], a longer duration of persistence was associated with reinitiation. Similarly, according to van Wijk et al. [16], the longer the patients had been on antihypertensive therapy, the more likely were the patients to restart treatment.

Administration of statins was associated with a lower likelihood of subsequent discontinuation among reinitiating patients. Statins are also used in secondary prevention in PAD patients [6,7]. It may be expected that patients taking statins are aware of the significance of secondary PAD prevention and adhere to both statin and ACEI/ARB treatments.

Administration of antiplatelet agents was associated with a higher probability of subsequent discontinuation of ACEIs/ARBs, but only in the subgroup of reinitiating new users. As mentioned above, being a new user was associated with an increased probability of subsequent discontinuation of ACEIs/ARBs. It is possible that the awareness of the significance of ACEI/ARB treatment in new users is low also in the case that these patients are using other medication (antiplatelet agents) indicated for secondary prevention of PAD [6,7]. On the other hand, Si et al. [14] reported a lower discontinuation of blood pressure-lowering agents in patients who were on antiplatelet or anticoagulant therapy. However, their study was focused on initial discontinuation, whereas our study analysed subsequent discontinuation after reinitiation, and the factors may have different associations with these two different events.

Administration of irbesartan was associated with a higher likelihood of reinitiation only in the subgroup of new users. Ramipril and losartan administration increased the likelihood of subsequent discontinuation only in the subgroup of new users. Unfortunately, the design of our study does not make it possible to explain these findings. Elliott et al. [20] reported valsartan being associated with a significantly decreased risk of discontinuation in comparison with hydrochlorothiazide, amlodipine, and lisinopril. In their retrospective longitudinal analysis, they evaluated a 1-year persistence and adherence to the monotherapy of different antihypertensives. In addition, in the retrospective observational study by Wogen et al. [21], significantly more patients receiving valsartan were persistent with treatment at 12 months after their first prescription in comparison with those taking amlodipine or lisinopril.

Our study has certain limitations that should be taken into consideration when interpreting the study findings. The database applied in the study was not primarily developed for research but for health insurance and reimbursement purposes. This database does not make it possible to differentiate who (i.e., the physician or the patient) decided on the discontinuation of ACEIs/ARBs. It is also impossible to determine whether medications were used as prescribed. On the other hand, the large sample size covering geographically the whole of Slovakia, as well as detailed and accurate data on patients´ comorbid conditions and medications, are the strengths of our study [12].

## 5. Conclusions

Reinitiation does not bring a resolution to the issue of non-persistence with ACEI/ARB treatment in older hypertensive patients with PAD, since almost a half of reinitiating patients discontinued ACEI/ARB treatment again. Patients need also education and support when reinitiating treatment in order to continue medication use. Being a new user was not associated with the probability of reinitiation, but was associated with an increased likelihood of the subsequent discontinuation in reinitiating patients. There were also other differences in factors associated with the reinitiation and subsequent discontinuation of ACEI/ARB treatment between new and prevalent users, and the user status at PAD diagnosis is thus also associated with medication use after initial discontinuation after diagnosis. In clinical practice, factors identified in our study may help identify patients with a decreased probability of reinitiation and an increased likelihood of subsequent discontinuation, who require special attention with a view to improving their persistence in order to achieve the beneficial effects of secondary prevention with ACEIs/ARBs.

## Figures and Tables

**Table 1:** Baseline characteristics of the study cohorts.

	Initially Non-Persistent (*n* = 1642)			Reinitiators (*n* = 875)		
Factor	Without Reinitiation (*n* = 767)	Reinitiators (*n* = 875)	*p*	Persistent (*n* = 461)	Non-Persistent (*n* = 414)	*p*
*Socio-demographic characteristics*						
Age	74.3 ± 6.4	73.9 ± 6.0	0.241 *	74.0 ± 6.0	73.7 ± 6.0	0.482 *
Female sex	465 (60.6)	492 (56.2)	0.071	260 (56.4)	232 (56.0)	0.915
University education	51 (6.6)	66 (7.5)	0.483	38 (8.2)	28 (6.8)	0.408
Employed patients	37 (4.8)	46 (5.3)	0.689	21 (4.6)	25 (6.0)	0.326
*History of CV events* ^a^						
History of ischemic stroke	206 (26.9)	208 (23.8)	0.151	159 (34.5)	97 (23.4)	**<0.001**
History of TIA	90 (11.7)	92 (10.5)	0.432	70 (15.2)	46 (11.1)	0.076
History of MI	106 (13.8)	87 (9.9)	**0.015**	68 (14.8)	52 (12.6)	0.347
*CV events during non-persistence/the period of reinitiation*						
Ischemic stroke during non-persistence/the period of reinitiation	80 (10.4)	48 (5.5)	**<0.001**	31 (6.7)	25 (6.0)	0.679
TIA during non-persistence/the period of reinitiation	35 (4.6)	26 (3.0)	0.089	10 (2.2)	5 (1.2)	0.274
MI during non-persistence/the period of reinitiation	39 (5.1)	33 (3.8)	0.195	33 (7.2)	6 (1.4)	**<0.001**
*Comorbid conditions*						
Number of comorbid conditions	2.7 ± 1.6	2.5 ± 1.6	0.053 *	2.5 ± 1.7	2.5 ± 1.6	0.613 *
Chronic heart failure	54 (7.0)	58 (6.6)	0.741	34 (7.4)	24 (5.8)	0.349
Atrial fibrillation	95 (12.4)	108 (12.3)	0.979	54 (11.7)	54 (13.0)	0.550
Diabetes mellitus	298 (38.9)	301 (34.4)	0.061	160 (34.7)	141 (34.1)	0.840
Hypercholesterolemia	290 (37.8)	317 (36.2)	0.508	174 (37.7)	143 (34.5)	0.325
Dementia	41 (5.3)	55 (6.3)	0.418	30 (6.5)	25 (6.0)	0.775
Depression	94 (12.3)	103 (11.8)	0.763	55 (11.9)	48 (11.6)	0.877
Anxiety disorders	240 (31.3)	255 (29.1)	0.344	128 (27.8)	127 (30.7)	0.344
Parkinson’s disease	25 (3.3)	41 (4.7)	0.142	19 (4.1)	22 (5.3)	0.405
Epilepsy	17 (2.2)	28 (3.2)	0.223	14 (3.0)	14 (3.4)	0.772
Bronchial asthma/COPD	169 (22.0)	160 (18.3)	0.058	74 (16.1)	86 (20.8)	0.071
*ACEI/ARB-related characteristics*						
ACEI/ARB agent ^b^						
Perindopril	355 (46.3)	408 (46.6)	0.379	250 (54.2)	224 (54.1)	0.858
Lisinopril	44 (5.7)	41 (4.7)		9 (2.0)	14 (3.4)	
Ramipril	113 (14.7)	133 (15.2)		71 (15.4)	55 (13.3)	
Enalapril	7 (0.9)	4 (0.5)		0 (0.0)	2 (0.5)	
Spirapril	1 (0.1)	1 (0.1)		1 (0.2)	1 (0.2)	
Trandolapril	119 (15.5)	123 (14.1)		76 (16.5)	69 (16.7)	
Quinapril	43 (5.6)	63 (7.2)		26 (5.6)	18 (4.3)	
Imidapril	13 (1.7)	12 (1.4)		2 (0.4)	3 (0.7)	
Fosinopril	13 (1.7)	5 (0.6)		2 (0.4)	2 (0.5)	
Valsartan	24 (3.1)	33 (3.8)		9 (2.0)	11 (2.7)	
Losartan	11 (1.4)	19 (2.2)		6 (1.3)	6 (1.4)	
Telmisartan	10 (1.3)	18 (2.1)		5 (1.1)	7 (1.7)	
Candesartan	12 (1.6)	10 (1.1)		1 (0.2)	0 (0.0)	
Irbesartan	2 (0.3)	5 (0.6)		3 (0.7)	2 (0.5)	
Patient´s co-payment (EUR) ^c^	3.1 ± 2.8	3.0 ± 2.6	0.340 *	2.9 ± 2.4	3.1 ± 2.4	0.172 *
New ACEI/ARB agent user ^d^	70 (9.1)	121 (13.8)	**0.003**	57 (12.4)	64 (15.5)	0.186
General practitioner as index prescriber	593 (77.3)	660 (75.4)	0.370	357 (77.4)	303 (73.2)	0.145
*CV co-medication*						
Number of medications	7.7 ± 2.8	7.4 ± 3.0	**0.044** *	7.6 ± 2.9	7.2 ± 3.1	**0.028** *
Number of CV medications	4.7 ± 2.2	4.5 ± 2.2	**0.042** *	4.6 ± 2.2	4.5 ± 2.2	0.207 *
Antiplatelet agents	550 (71.7)	596 (68.1)	0.114	322 (69.8)	274 (66.2)	0.246
Anticoagulants	189 (24.6)	195 (22.3)	0.261	106 (23.0)	89 (21.5)	0.596
Cardiac glycosides	59 (7.7)	49 (5.6)	0.088	25 (5.4)	24 (5.8)	0.810
Antiarrhythmic agents	52 (6.8)	60 (6.9)	0.950	26 (5.6)	34 (8.2)	0.133
Beta-blockers	129 (16.8)	158 (18.1)	0.510	90 (19.5)	68 (16.4)	0.234
Thiazide diuretics	145 (18.9)	163 (18.6)	0.886	87 (18.9)	76 (18.4)	0.845
Loop diuretics	163 (21.3)	155 (17.7)	0.070	91 (19.7)	64 (15.5)	0.098
Mineralocorticoid receptor antagonists	56 (7.3)	50 (5.7)	0.192	32 (6.9)	18 (4.3)	0.099
Calcium channel blockers	210 (27.4)	216 (24.7)	0.214	117 (25.4)	99 (23.9)	0.615
Statins	543 (70.8)	650 (74.3)	0.113	362 (78.5)	288 (69.6)	0.002
Lipid-lowering agents other than statins ^e^	78 (10.2)	73 (8.3)	0.201	38 (8.2)	35 (8.5)	0.910
Duration of persistence/non-persistence (months) ^f^	25.4 ± 16.1	15.5 ± 13.7	**<0.001** *	17.3 ± 13.1	15.0 ± 9.5	0.485 *

In the case of categorical variables, values represent the frequency, and the percentages are provided in parentheses (% of *n*). In the case of continuous variables, means ± standard deviations are provided. TIA—transient ischemic attack; MI—myocardial infarction; COPD—chronic obstructive pulmonary disease; CV—cardiovascular; p—statistical significance according to the χ^2^-test; * statistical significance according to the Mann–Whitney U test; in the case of statistically significant results (*p* < 0.05), the values are expressed in bold. ^a^ The time period covered by “history”—5 years before the index date of the analysis of reinitiation/analysis of subsequent discontinuation after reinitiation. ^b^ ACEI/ARB agent—in the analysis of reinitiation: the last ACEI/ARB agent before initial discontinuation in our previous study [12]/in the analysis of subsequent discontinuation after reinitiation: the ACEI/ARB agent administered initially at the time of reinitiation. ^c^ Patient´s co-payment–calculated as the cost of ACEI/ARB treatment paid by the patient per month; in the analysis of reinitiation: co-payment for the last ACEI/ARB agent before initial discontinuation in our previous study [12]/in the analysis of subsequent discontinuation after reinitiation: co-payment for the ACEI/ARB agent administered initially at the time of reinitiation. ^d^ New ACEI/ARB agent user—patient in whom ACEI/ARB treatment was initiated in association with the diagnosis of peripheral arterial disease. ^e^ Lipid-lowering agents other than statins—ezetimibe and fibrates. ^f^ In the analysis of reinitiation: duration of persistence before initial discontinuation in our previous study [12]/in the analysis of subsequent discontinuation after reinitiation: duration of the period of non-persistence (before reinitiation).

## Data Availability

The data that support the findings of this study are available from the General Health Insurance Company, but restrictions apply to the availability of these data which were used under a license for the present study and are thus not publicly available. Data are, however, available from the authors upon reasonable request and subject to permission of the General Health Insurance Company.

## Supplementary Materials

The following are available online at https://www.mdpi.com/article/10.3390/biomedicines11020368/s1; Table S1: Baseline characteristics of the cohort used in the analysis of reinitiation among prevalent and new users; Table S2: Baseline characteristics of the cohort used in the analysis of the subsequent discontinuation after reinitiation among prevalent and new users.
