# Supplementary material for: Reinitiation and Subsequent Discontinuation of Angiotensin-Converting Enzyme Inhibitors and Angiotensin Receptor Blockers among New and Prevalent Users Aged 65 Years or More with Peripheral Arterial Disease
Source: Biomedicines. 2023 Jan 26;11(2):368. doi: 10.3390/biomedicines11020368 (PMC9953445; doi:10.3390/biomedicines11020368)
Supplement: Supplementary file 1 [file biomedicines-11-00368-s001.zip › Supplementary Table S1.pdf]

Supplementary Table S1. Baseline characteristics of the cohort used in the analysis of reinitiation among prevalent and new users.

Factor	Prevalent users				New users			
	All (n = 1451)	Without reinitiation (n = 697)	With reinitiation (n = 754)	p	All (n = 191)	Without reinitiation (n = 70)	With reinitiation (n = 121)	p
Socio-demographic characteristics								
Age	74.2 ± 6.2	74.4 ± 6.4	74.1 ± 6.0	0.428**	72.9 ± 5.7	73.3 ± 5.6	72.7 ± 5.8	0.379**
Female sex	850 (58.6)	424 (60.8)	426 (56.5)	0.094	107 (56.0)	41 (58.6)	66 (54.5)	0.589
University education	102 (7.0)	47 (6.7)	55 (7.3)	0.682	15 (7.9)	4 (5.7)	11 (9.1)	0.403
Employed patients	69 (4.8)	33 (4.7)	36 (4.8)	0.971	14 (7.3)	4 (5.7)	10 (8.3)	0.515
History of CV events[a]								
History of ischemic stroke	379 (26.1)	194 (27.8)	185 (24.5)	0.153	35 (18.3)	12 (17.1)	23 (19.0)	0.748
History of TIA	161 (11.1)	81 (11.6)	80 (10.6)	0.540	21 (11.0)	9 (12.9)	12 (9.9)	0.531
History of MI	172 (11.9)	98 (14.1)	74 (9.8)	**0.012**	21 (11.0)	8 (11.4)	13 (10.7)	0.884
CV events during non-persistence								
Ischemic stroke during non-persistence	106 (7.3)	68 (9.8)	38 (5.0)	**<0.001**	22 (11.5)	12 (17.1)	10 (8.3)	0.064
TIA during non-persistence	52 (3.6)	31 (4.4)	21 (2.8)	0.089	9 (4.7)	4 (5.7)	5 (4.1)	0.727*
MI during non-persistence	63 (4.3)	36 (5.2)	27 (3.6)	0.139	9 (4.7)	3 (4.3)	6 (5.0)	1.000*
Comorbid conditions								
Number of comorbid conditions	2.7 ± 1.6	2.7 ± 1.6	2.6 ± 1.6	0.127**	1.9 ± 1.6	1.9 ± 1.5	1.9 ± 1.7	0.720**
Chronic heart failure	98 (6.8)	49 (7.0)	49 (6.5)	0.687	14 (7.3)	5 (7.1)	9 (7.4)	0.940
Atrial fibrillation	192 (13.2)	89 (12.8)	103 (13.7)	0.617	11 (5.8)	6 (8.6)	5 (4.1)	0.204
Diabetes mellitus	540 (37.2)	281 (40.3)	259 (34.4)	**0.019**	59 (30.9)	17 (24.3)	42 (34.7)	0.133

(Table continued)

Factor	Prevalent users: All (n = 1451)	Prevalent users: Without reinitiation (n = 697)	Prevalent users: With reinitiation (n = 754)	p	New users: All (n = 191)	New users: Without reinitiation (n = 70)	New users: With reinitiation (n = 121)	p
Hypercholesterolemia	562 (38.7)	273 (39.2)	289 (38.3)	0.743	45 (23.6)	17 (24.3)	28 (23.1)	0.857
Dementia	90 (6.2)	39 (5.6)	51 (6.8)	0.357	6 (3.1)	2 (2.9)	4 (3.3)	1.000*
Depression	181 (12.5)	89 (12.8)	92 (12.2)	0.744	16 (8.4)	5 (7.1)	11 (9.1)	0.640
Anxiety disorders	446 (30.7)	220 (31.6)	226 (30.0)	0.512	49 (25.7)	20 (28.6)	29 (24.0)	0.483
Parkinson's disease	59 (4.1)	25 (3.6)	34 (4.5)	0.374	7 (3.7)	0 (0.0)	7 (5.8)	**0.049***
Epilepsy	44 (3.0)	16 (2.3)	28 (3.7)	0.116	1 (0.5)	1 (1.4)	0 (0.0)	0.366*
Bronchial asthma/COPD	298 (20.5)	155 (22.2)	143 (19.0)	0.123	31 (16.2)	14 (20.0)	17 (14.0)	0.283
ACEI/ARB related characteristics								
ACEI/ARB agent[b]								
Perindopril	664 (45.8)	321 (46.1)	343 (45.5)	0.347	99 (51.8)	34 (48.6)	65 (53.7)	0.395
Lisinopril	76 (5.2)	43 (6.2)	33 (4.4)		9 (4.7)	1 (1.4)	8 (6.6)	
Ramipril	211 (14.5)	97 (13.9)	114 (15.1)		35 (18.3)	16 (22.9)	19 (15.7)	
Enalapril	11 (0.8)	7 (1.0)	4 (0.5)		0 (0.0)	0 (0.0)	0 (0.0)	
Spirapril	2 (0.1)	1 (0.1)	1 (0.1)		0 (0.0)	0 (0.0)	0 (0.0)	
Trandolapril	210 (14.5)	105 (15.1)	105 (13.9)		32 (16.8)	14 (20.0)	18 (14.9)	
Quinapril	98 (6.8)	40 (5.7)	58 (7.7)		8 (4.2)	3 (4.3)	5 (4.1)	
Imidapril	23 (1.6)	12 (1.7)	11 (1.5)		2 (1.0)	1 (1.4)	1 (0.8)	
Fosinopril	18 (1.2)	13 (1.9)	5 (0.7)		0 (0.0)	0 (0.0)	0 (0.0)	
Valsartan	56 (3.9)	23 (3.3)	33 (4.4)		1 (0.5)	1 (1.4)	0 (0.0)	

(Table continued)

Factor	Prevalent users All (n = 1451)	Prevalent users Without reinitiation (n = 697)	Prevalent users With reinitiation (n = 754)	p	New users All (n = 191)	New users Without reinitiation (n = 70)	New users With reinitiation (n = 121)	p
Losartan	29 (2.0)	11 (1.6)	18 (2.4)		1 (0.5)	0 (0.0)	1 (0.8)	
Telmisartan	27 (1.9)	10 (1.4)	17 (2.3)		1 (0.5)	0 (0.0)	1 (0.8)	
Candesartan	22 (1.5)	12 (1.7)	10 (1.3)		0 (0.0)	0 (0.0)	0 (0.0)	
Irbesartan	4 (0.3)	2 (0.3)	2 (0.3)		3 (1.6)	0 (0.0)	3 (2.5)	
Patient´s co-payment (EUR)[c]	3.8 ± 2.5	3.8 ± 2.6	3.7 ± 2.5	0.530**	3.7 ± 2.2	3.5 ± 2.1	3.5 ± 2.4	0.412**
General practitioner as index prescriber	1137 (78.4)	550 (78.9)	587 (77.9)	0.625	116 (60.7)	43 (61.4)	73 (60.3)	0.881
CV co-medication								
Number of medications	7.7 ± 2.8	7.8 ± 2.7	7.6 ± 2.9	0.286**	6.1 ± 3.3	6.8 ± 3.4	5.6 ± 3.3	**0.049****
Number of CV medications	4.7 ± 2.2	4.8 ± 2.2	4.7 ± 2.2	0.197**	3.9 ± 2.1	4.1 ± 2.0	3.8 ± 2.1	0.163**
Antiplatelet agents	1014 (69.9)	498 (71.4)	516 (68.4)	0.211	132 (69.1)	52 (74.3)	80 (66.1)	0.239
Anticoagulants	348 (24.0)	171 (24.5)	177 (23.5)	0.637	36 (18.8)	18 (25.7)	18 (14.9)	0.065
Cardiac glycosides	99 (6.8)	56 (8.0)	43 (5.7)	0.078	9 (4.7)	3 (4.3)	6 (5.0)	1.000*
Antiarrhythmic agents	105 (7.2)	49 (7.0)	56 (7.4)	0.771	7 (3.7)	3 (4.3)	4 (3.3)	0.708*
Beta-blockers	264 (18.2)	122 (17.5)	142 (18.8)	0.512	23 (12.0)	7 (10.0)	16 (13.2)	0.510
Thiazide diuretics	287 (19.8)	138 (19.8)	149 (19.8)	0.986	21 (11.0)	7 (10.0)	14 (11.6)	0.738
Loop diuretics	292 (20.1)	153 (22.0)	139 (18.4)	0.095	26 (13.6)	10 (14.3)	16 (13.2)	0.837
Mineralocorticoid receptor antagonists	99 (6.8)	53 (7.6)	46 (6.1)	0.257	7 (3.7)	3 (4.3)	4 (3.3)	0.708*
Calcium channel blockers	401 (27.6)	198 (28.4)	203 (26.9)	0.528	25 (13.1)	12 (17.1)	13 (10.7)	0.206
Statins	1050 (72.4)	493 (70.7)	557 (73.9)	0.181	143 (74.9)	50 (71.4)	93 (76.9)	0.404

(Table continued)

Factor	Prevalent users All (n = 1451)	Prevalent users Without reinitiation (n = 697)	Prevalent users With reinitiation (n = 754)	p	New users All (n = 191)	New users Without reinitiation (n = 70)	New users With reinitiation (n = 121)	p
Lipid-lowering agents other than statins[d]	140 (9.6)	74 (10.6)	66 (8.8)	0.230	11 (5.8)	4 (5.7)	7 (5.8)	1.000*
Duration of persistence (months)[e]	21.1 ± 15.7	26.3 ± 15.9	16.3 ± 13.8	**<0.001****	12.9 ± 10.8	17.0 ± 14.9	10.5 ± 9.2	**<0.001****

In the case of categorical variables, values represent the frequency and the percentages are provided in parentheses (% of n). In the case of continuous variables, means ± standard deviations are provided. TIA – transient ischemic attack; MI – myocardial infarction; COPD – chronic obstructive pulmonary disease; CV – cardiovascular; p – statistical significance according to the χ^2-test; * statistical significance according to the Fisher exact test; ** statistical significance according to the Mann-Whitney U test; in the case of statistically significant results (p<0.05), the values are expressed in bold. [a]The time period covered by "history" – 5 years before the index date of the analysis of reinitiation. [b]ACEI/ARB agent – the last ACEI/ARB agent before the initial discontinuation. [c]Patient´s co-payment – calculated as the cost of ACEI/ARB treatment paid by the patient per month; co-payment for the last ACEI/ARB agent before the initial discontinuation. [d]Lipid lowering agents other than statins – ezetimibe and fibrates. [e]Duration of the period of persistence before the initial discontinuation.
