# Supplementary material for: Reinitiation and Subsequent Discontinuation of Angiotensin-Converting Enzyme Inhibitors and Angiotensin Receptor Blockers among New and Prevalent Users Aged 65 Years or More with Peripheral Arterial Disease
Source: Biomedicines. 2023 Jan 26;11(2):368. doi: 10.3390/biomedicines11020368 (PMC9953445; doi:10.3390/biomedicines11020368)
Supplement: Supplementary file 1 [file biomedicines-11-00368-s001.zip › Supplementary Table S2.pdf]

Supplementary Table S2. Baseline characteristics of the cohort used in the analysis of the subsequent discontinuation after reinitiation among prevalent and new users.

Factor	Prevalent users				New users			
	All (n = 754)	Persistent (n = 404)	Non-persistent (n = 350)	p	All (n = 121)	Persistent (n = 57)	Non-persistent (n = 64)	p
Socio-demographic characteristics								
Age	74.1 ± 6.0	74.1 ± 6.1	74.0 ± 6.0	0.975**	72.7 ± 5.8	73.4 ± 5.6	72.1 ± 6.0	0.114**
Female sex	426 (56.5)	228 (56.4)	198 (56.6)	0.970	66 (54.5)	32 (56.1)	34 (53.1)	0.740
University education	55 (7.3)	33 (8.2)	22 (6.3)	0.321	11 (9.1)	5 (8.8)	6 (9.4)	0.908
Employed patients	36 (4.8)	16 (4.0)	20 (5.7)	0.260	10 (8.3)	5 (8.8)	5 (7.8)	0.848
History of CV events[a]								
History of ischemic stroke	223 (29.6)	142 (35.1)	81 (23.1)	**<0.001**	33 (27.3)	17 (29.8)	16 (25.0)	0.552
History of TIA	99 (13.1)	63 (15.6)	36 (10.3)	**0.031**	17 (14.0)	7 (12.3)	10 (15.6)	0.597
History of MI	101 (13.4)	58 (14.4)	43 (12.3)	0.405	19 (15.7)	10 (17.5)	9 (14.1)	0.599
CV events during the period of reinitiation								
Ischemic stroke during the period of reinitiation	51 (6.8)	27 (6.7)	24 (6.9)	0.924	5 (4.1)	4 (7.0)	1 (1.6)	0.187*
TIA during the period of reinitiation	14 (1.9)	9 (2.2)	5 (1.4)	0.418	1 (0.8)	1 (1.8)	0 (0.0)	0.471*
MI during the period of reinitiation	33 (4.4)	28 (6.9)	5 (1.4)	**<0.001**	6 (5.0)	5 (8.8)	1 (1.6)	0.099*
Comorbid conditions								
Number of comorbid conditions	2.6 ± 1.6	2.6 ± 1.6	2.6 ± 1.5	0.805**	1.9 ± 1.6	1.7 ± 1.5	2.1 ± 1.6	0.166**
Chronic heart failure	49 (6.5)	30 (7.4)	19 (5.4)	0.267	9 (7.4)	4 (7.0)	5 (7.8)	1.000*
Atrial fibrillation	103 (13.7)	52 (12.9)	51 (14.6)	0.498	5 (4.1)	2 (3.5)	3 (4.7)	1.000*

(Table continued)

Factor	Prevalent users				New users			
	**All (n = 754)**	**Persistent (n = 404)**	**Non-persistent (n = 350)**	**p**	**All (n = 121)**	**Persistent (n = 57)**	**Non-persistent (n = 64)**	**p**
Diabetes mellitus	259 (34.4)	144 (35.6)	115 (32.9)	0.422	42 (34.7)	16 (28.1)	26 (40.6)	0.148
Hypercholesterolemia	289 (38.3)	163 (40.3)	126 (36.0)	0.221	28 (23.1)	11 (19.3)	17 (26.6)	0.344
Dementia	51 (6.8)	29 (7.2)	22 (6.3)	0.626	4 (3.3)	1 (1.8)	3 (4.7)	0.621*
Depression	92 (12.2)	47 (11.6)	45 (12.9)	0.609	11 (9.1)	8 (14.0)	3 (4.7)	0.074
Anxiety disorders	226 (30.0)	115 (28.5)	111 (31.7)	0.331	29 (24.0)	13 (22.8)	16 (25.0)	0.778
Parkinson's disease	34 (4.5)	16 (4.0)	18 (5.1)	0.435	7 (5.8)	3 (5.3)	4 (6.3)	1.000*
Epilepsy	28 (3.7)	14 (3.5)	14 (4.0)	0.699	0 (0.0)	0 (0.0)	0 (0.0)	
Bronchial asthma/COPD	143 (19.0)	70 (17.3)	73 (20.9)	0.217	17 (14.0)	4 (7.0)	13 (20.3)	**0.036**
ACEI/ARB related characteristics								
ACEI/ARB agent[b]								
Perindopril	405 (53.7)	215 (53.2)	190 (54.3)	0.726	69 (57.0)	35 (61.4)	34 (53.1)	0.312
Lisinopril	20 (2.7)	8 (2.0)	12 (3.4)		3 (2.5)	1 (1.8)	2 (3.1)	
Ramipril	108 (14.3)	66 (16.3)	42 (12.0)		18 (14.9)	5 (8.8)	13 (20.3)	
Enalapril	2 (0.3)	0 (0.0)	2 (0.6)		0 (0.0)	0 (0.0)	0 (0.0)	
Spirapril	2 (0.3)	1 (0.2)	1 (0.3)		0 (0.0)	0 (0.0)	0 (0.0)	
Trandolapril	117 (15.5)	60 (14.9)	57 (16.3)		28 (23.1)	16 (28.1)	12 (18.8)	
Quinapril	43 (5.7)	26 (6.4)	17 (4.9)		1 (0.8)	0 (0.0)	1 (1.6)	
Imidapril	5 (0.7)	2 (0.5)	3 (0.9)		0 (0.0)	0 (0.0)	0 (0.0)	
Fosinopril	4 (0.5)	2 (0.5)	2 (0.6)		0 (0.0)	0 (0.0)	0 (0.0)	

(Table continued)

Factor	Prevalent users All (n = 754)	Prevalent users Persistent (n = 404)	Prevalent users Non-persistent (n = 350)	Prevalent users p	New users All (n = 121)	New users Persistent (n = 57)	New users Non-persistent (n = 64)	New users p
Valsartan	20 (2.7)	9 (2.2)	11 (3.1)		0 (0.0)	0 (0.0)	0 (0.0)	
Losartan	11 (1.5)	6 (1.5)	5 (1.4)		1 (0.8)	0 (0.0)	1 (1.6)	
Telmisartan	11 (1.5)	5 (1.2)	6 (1.7)		1 (0.8)	0 (0.0)	1 (1.6)	
Candesartan	1 (0.1)	1 (0.2)	0 (0.0)		0 (0.0)	0 (0.0)	0 (0.0)	
Irbesartan	5 (0.7)	3 (0.7)	2 (0.6)		0 (0.0)	0 (0.0)	0 (0.0)	
Patient´s co-payment (EUR)[c]	3.0 ± 2.7	2.9 ± 2.5	3.1 ± 2.7	0.349**	2.9 ± 2.3	2.4 ± 1.7	3.4 ± 2.5	0.176**
General practitioner as index prescriber	587 (77.9)	322 (79.7)	265 (75.7)	0.188	73 (60.3)	35 (61.4)	38 (59.4)	0.820
CV co-medication								
Number of medications	7.6 ± 2.9	7.8 ± 2.8	7.4 ± 3.0	0.055**	5.6 ± 3.3	5.6 ± 3.4	5.7 ± 3.2	0.931**
Number of CV medications	4.7 ± 2.2	4.7 ± 2.1	4.6 ± 2.2	0.201**	3.8 ± 2.1	3.8 ± 2.3	3.8 ± 2.0	0.579**
Antiplatelet agents	516 (68.4)	284 (70.3)	232 (66.3)	0.237	80 (66.1)	38 (66.7)	42 (65.6)	0.904
Anticoagulants	177 (23.5)	96 (23.8)	81 (23.1)	0.841	18 (14.9)	10 (17.5)	8 (12.5)	0.436
Cardiac glycosides	43 (5.7)	22 (5.4)	21 (6.0)	0.743	6 (5.0)	3 (5.3)	3 (4.7)	1.000*
Antiarrhythmic agents	56 (7.4)	25 (6.2)	31 (8.9)	0.163	4 (3.3)	1 (1.8)	3 (4.7)	0.621*
Beta-blockers	142 (18.8)	81 (20.0)	61 (17.4)	0.359	16 (13.2)	9 (15.8)	7 (10.9)	0.432
Thiazide diuretics	149 (19.8)	80 (19.8)	69 (19.7)	0.976	14 (11.6)	7 (12.3)	7 (10.9)	0.818
Loop diuretics	139 (18.4)	85 (21.0)	54 (15.4)	**0.048**	16 (13.2)	6 (10.5)	10 (15.6)	0.409
Mineralocorticoid receptor antagonists	46 (6.1)	30 (7.4)	16 (4.6)	0.102	4 (3.3)	2 (3.5)	2 (3.1)	1.000*
Calcium channel blockers	203 (26.9)	114 (28.2)	89 (25.4)	0.389	13 (10.7)	3 (5.3)	10 (15.6)	0.066
Statins	557 (73.9)	316 (78.2)	241 (68.9)	**0.004**	93 (76.9)	46 (80.7)	47 (73.4)	0.344

(Table continued)

Factor	Prevalent users All (n = 754)	Prevalent users Persistent (n = 404)	Prevalent users Non-persistent (n = 350)	Prevalent users p	New users All (n = 121)	New users Persistent (n = 57)	New users Non-persistent (n = 64)	New users p
Lipid-lowering agents other than statins[d]	66 (8.8)	34 (8.4)	32 (9.1)	0.725	7 (5.8)	4 (7.0)	3 (4.7)	0.706*
Duration of non-persistence (months)[e]	15.7 ± 11.2	16.7 ± 12.8	14.5 ± 9.0	0.425**	19.4 ± 13.1	21.0 ± 14.6	17.9 ± 11.5	0.670**

In the case of categorical variables, values represent the frequency and the percentages are provided in parentheses (% of n). In the case of continuous variables, means ± standard deviations are provided. TIA – transient ischemic attack; MI – myocardial infarction; COPD – chronic obstructive pulmonary disease; CV – cardiovascular; p – statistical significance according to the χ^2-test; * statistical significance according to the Fisher exact test; ** statistical significance according to the Mann-Whitney U test; in the case of statistically significant results ($p<0.05$), the values are expressed in bold. [a]The time period covered by "history" – 5 years before the index date of the analysis of the subsequent discontinuation after reinitiation. [b]ACEI/ARB agent – the ACEI/ARB agent administered initially at the time of reinitiation. [c]Patient´s co-payment – calculated as the cost of ACEI/ARB treatment paid by the patient per month; co-payment for the ACEI/ARB agent administered initially at the time of reinitiation. [d]Lipid lowering agents other than statins – ezetimibe and fibrates. [e]Duration of the period of non-persistence before reinitiation.
